# Research Note: A validation of an image-based method to estimate chicken comb size

**DOI:** 10.1016/j.psj.2024.103434

**Published:** 2024-01-06

**Authors:** Klara J. Grethen, Laura Candelotto, Yamenah Gómez, Michael J. Toscano

**Affiliations:** ⁎ Division of Animal Welfare, Veterinary Public Health Institute, Center for Proper Housing: Poultry and Rabbits (ZTHZ), University of Bern, Zollikofen 3052, Switzerland; † Graduate School of Cellular and Biomedical Sciences, University of Bern, Bern 3012, Switzerland

**Keywords:** comb size, image analysis, area measure, validation

## Abstract

Chicken combs carry important information about the individual, especially the size has been related to sexual reproduction, health, and social signaling. Comb size is usually estimated by weighing removed combs or by calculating the product of the comb's longest and highest dimensions (**LHA**) to approximate comb area based on measures of a ruler or caliper. These methods have several shortcomings including invasiveness or imprecision. As a result, more recent efforts have employed pixel-based approximations of comb area (**PBA**) from images. However, the validity of PBA to estimate comb area and how the approximation compares to previous approximation methods, such as LHA, is unknown. Therefore, we developed an apparatus for taking standardized images of the head position of the hens and then applied PBA using the software ImageJ. The hens were each photographed 3 times by 2 different handlers. We first tested the accuracy of the pixel-based area approximation on 3 geometric shapes of known area. Second, we tested the precision of PBA of 15 hens (Dekalb White), evaluated as within-image and within-individual hen precision. Furthermore, we compared the PBA with the LHA based on measures of a caliper. The PBA was both accurate and precise, whereas the LHA overestimated comb area with increasing overestimation for larger combs. Due to the greater accuracy of the PBA, as well as future possibilities of automation and inclusion of further measures, we suggest PBA as a more reliable approach to estimate comb area than LHA. Additionally, our results demonstrate that the outcomes of LHA should be evaluated on an ordinal scale level only.

## INTRODUCTION

Body features of animals can relay important information of an individual's taxon, gender, age, health, or position within a group (Zelditch et al., 2012). Within commercial chickens (*Gallus gallus domesticus*), an example of such a feature is the comb, which serves multiple functions ranging from temperature regulation to social signaling (Mukhtar and Khan, 2012). Specifically, the size is related in both modern day chickens and their ancestors to androgen concentrations, reproductive success (Zuk et al., 1995; Joseph et al., 2003; Navara et al., 2012), health status (Zuk et al., 1990), dominance (D'Eath and Keeling, 2003; O'Connor et al., 2011), and recognition of conspecifics (Guhl and Ortman, 1953).

**Figure 1:**
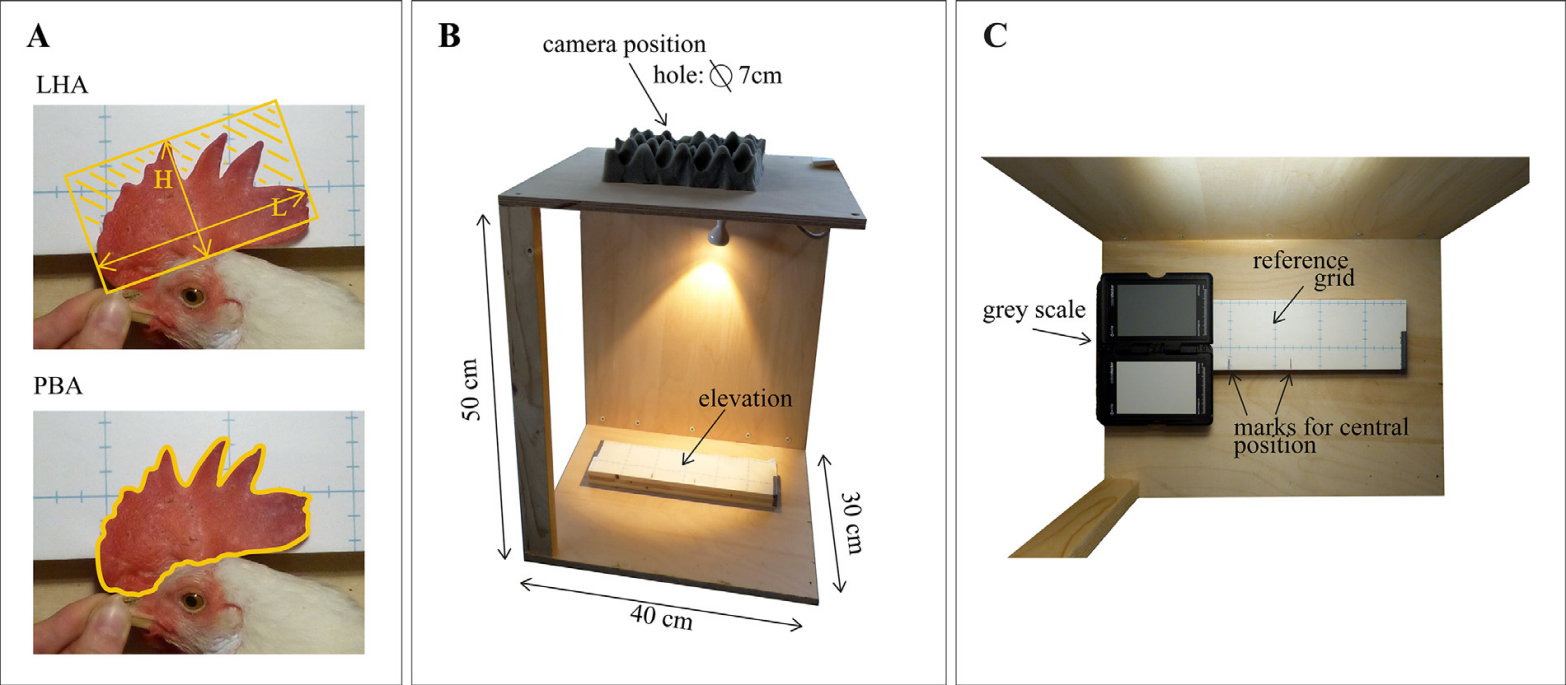
Illustration of comb area approximations and imaging apparatus. In panel A, the estimated comb area, based on 2 different methods, is depicted by a yellow outline. In the top image, the LHA (product of length [L] and height [H]) is shown. The striped area visualizes the expected overestimation of area when using LHA. In the bottom image, the PBA (pixel-based approximation of comb area) is shown. In panel B, the full apparatus is depicted from a side perspective. Panel C shows the camera field of view with a grey scale added as color reference for image color constancy.

One method in research to estimate the comb size is to weigh the comb after postmortem removal (Jones and Lamoreux, 1943; Moro et al., 2015). The comb weight is the most accurate representation of comb size, as it captures the entire volume of the body feature, but the method is only compatible with procedures where the hen is killed. Alternatively, the comb size is estimated by directly measuring either or both the longest and highest dimension of the comb using a ruler or caliper (Zuk et al., 1990, 1995; Navara et al., 2012). Depending on how the points of measurement for the longest and highest dimensions are defined, the simple length (**L**) and height (**H**) measures can vary greatly. As an additional drawback, each measure only captures one dimension of the 3-dimensional feature. Another option to estimate comb size is to multiply the comb's L and H, as an approximation of the comb area (**LHA**) (D'Eath and Keeling, 2003; Joseph et al., 2003; O'Connor et al., 2011). The LHA was deemed superior to the use of single dimensions early on (Jones and Lamoreux, 1943) due to its simplicity, speed, and high correlation with the comb weight, which likely led to its continued use in research to the present. However, the LHA results in an overestimation of the comb area, as the saw-tooth shape of the comb is not taken into account (Figure 1A), and thus remains a questionable gold standard.

Advances in image analysis using software that provides pixel-based approximations of the comb area (**PBA**) or geometric morphometric calculations potentially allow for more precise estimates over direct measurements such as the LHA. Image-based methods have already been applied to estimate the area of chicken combs (Navara et al., 2012; Moro et al., 2015) and pixel-based approximations of comb H and area have been shown to correlate well with the comb H measured directly with a ruler (Joseph et al., 2003). However, PBA has not been validated with respect to its accuracy and precision. Therefore, the added benefit of image-based methods is not clear, considering their increased complexity in application, especially if direct methods provide adequate estimates. For the reasons stated above, we hypothesized that the use of image-based methods by means of pixel-based approximation (i.e., PBA), would likely result in an accurate and precise comb area estimate which would be superior to the LHA.

To test the hypothesis, we developed a simple, standardized protocol for comb area estimation from images which were compared against values measured with a caliper. We first validated the pixel-based method, by testing the accuracy on geometric shapes of known areas. The precision of PBA was tested on actual chicken combs by taking repeated images of the same combs and positioning animals using different handlers. Second, the PBA was compared to the LHA. We did not consider pixel-based approximations of comb L and H, as they have already been assessed in a previous study (Joseph et al., 2003) and 1-dimensional measures are inferior to 2-dimensional measures in representing a 3-dimensional feature. We did assess whether L or H explained more variation of the PBA. Finally, we discussed the advantages and disadvantages of both image-based and direct methods.

## MATERIAL AND METHODS

### Animal Husbandry

The study was approved by the cantonal authority of Bern (BE75/19) and conducted according to national ethics regulations.

We housed 225 laying hens (Dekalb White) in a single pen of a semi-commercial barn (containing 19 additional replicate pens that were not used) at the Aviforum facilities in Zollikofen, Switzerland. The pen contained a Vencomatic Bolegg Terrace aviary system (Krieger AG, Ruswil, Switzerland) that gave hens access to a covered outdoor area. Water and feed were available ad libitum. To estimate comb area with the 2 approximation methods (i.e., LHA and PBA), we randomly selected 15 individuals at 47 weeks of age. The sample size of 15 hens was chosen as the smallest possible representative sample, utilizing repeated measures, assuming low within-individual variation.

All LHA and PBA measures were taken outside the pen during one single session lasting less than 60 min in total. All hens were caught and crated according to standard procedures for health or phenotypic assessments, then each bird was handled for the measurements for less than 5 min. Therefore, no prior habituation to the process was conducted in order to keep catching events and consequent stress to a minimum.

### Direct (Caliper) Method

We used a standard, electronic digital caliper (150 mm, resolution: 0.1 mm/0.01″, accuracy: ± 0.1 mm/0.01″) to measure the comb L and H (Figure 1A). For L, we measured the distance between the most anterior point of the comb (i.e., closest to the beak) to the most posterior point on the last spike. For H, the measurement was taken from the most superior point of the skull to the tip of the highest reaching spike of the comb. All measures were performed by the same person. The values of L and H were then multiplied to calculate LHA.

### Pixel-Based Method

#### Apparatus

To standardize the procedure of taking images, we constructed a wooden, rectangular frame (Figure 1B). A digital compact camera (Lumix DMC-FZ150, Panasonic Rotkreuz, Switzerland) with external release button was fixed in a foam cushion on the top of the frame at a height of 50 cm from the bottom of the apparatus. Hens were placed in the frame with their comb on top of an elevated block (height: 2.7 cm) (Figures 1A and 1C) to reduce contortions of the comb shape. A reference grid (containing square dimensions: 5 × 5 cm, Figure 1C) was fastened to the block for the calibration of pixel dimensions during the image analysis (see “Image processing”). To minimize perspective distortion, we marked a central position for placement of the combs on the reference grid that represented the center of the camera's field of view (Figure 1C). We added a lamp to the apparatus which provided constant lighting conditions and removed the need for a flash.

#### Image Collection

For the validation of the PBA, we photographed 3 distinct geometric shapes of known areas (square = 29.5 cm², triangle = 18 cm², rectangle = 50.15 cm²) to evaluate measurement accuracy (i.e., how close the measured value is to the true value). Each geometric shape was photographed 3 times in random positions within the marked area of the central position on the reference grid of the apparatus (total of 9 images).

We photographed combs of 15 hens to test the precision (i.e., the repeatability of measures) of PBA. Each hen was placed into the frame by one of 2 distinct handlers and photographed. For the placement of the hen, the handler held the hen's legs close to the bird's body and lowered the hen onto the side of the comb's natural drop direction (if the comb dropped to the right side of the hen's face, on her right side, and vice versa). When the hen was positioned, her beak was gripped by the handler with the index finger and thumb of one hand to fixate the head position with the comb on the elevated block for the picture. After the first picture, the hen was given to the second handler who placed the hen equally. Each handling event took less than 20 s. The hen was photographed 6 times, with 3 repeats per handler. For a final, seventh image, an image was taken with the hens facing the opposite direction (always placed by the same handler) to test the effect of the natural drop direction on precision of PBA.

#### Image Processing

All image processing was conducted by 1 person. To measure the area of the geometric shapes and combs using pixel-based approximation, the software ImageJ2 was applied to the images (Fiji distribution 2.0.0-rc-69/1.53c, Java 1.8.0_172, 64-bit) (Schindelin et al., 2012).

On the first image, the pixel scale was calibrated using the known dimension of the reference grid. The pixel scale was set by assessing the number of pixels covering the known distance of 5 cm of the reference grid, allowing the program to relate pixel number and size to cm². The scaling was done anew, whenever the apparatus (including camera) was repositioned. After the scale was set, the PBA was extracted for each image in 4 steps:

1. The image was cropped to show only the relevant features of the comb or geometric shape and the reference grid (Figure 1A).
2. If a hen had very red facial features or the wattles could not be cropped, these were manually color-blocked in gray using the paintbrush tool, as they would have interfered with the selection by color.
3. To select the comb or shape, a color threshold was adjusted in the CIELAB (L*a*b) color space to fit the comb or shape color (result example, see Figure 1A).
4. The area of the selection was then extracted using the inbuilt measure function of ImageJ2, which converted the number of pixels of the selected area into cm².

After all images had been processed, the resulting outcomes were saved for analysis.

### Data Analysis

All analyses were performed in R (v. 4.2.0). As the comb is reduced to a 2-dimensional object both for the LHA and PBA, we referred to *comb area* in the following instead of comb size.

To validate pixel-based approximations in terms of measurement accuracy, the estimated areas of 2-dimensional geometric shapes were evaluated for agreement with their known areas using Lin's Concordance Correlation Coefficient (**CCC**) (Lin, 1989) (package “DescTools,” v. 0.99.45). A CCC < 0.9 was considered poor and > 0.99 excellent (McBride, 2005). We calculated the mean absolute percentage error (MAPE = $mean\left(\frac{\left|\text{known}\;\text{area}\;-\;\text{measured}\;\text{area}\right|}{\text{known}\;\text{area}}*100\right)$) and the mean absolute differences (mean(|knownarea−measuredarea|)) across the 9 images of the geometric shapes as an estimate of measurement error. A MAPE of below 1% was accepted as excellent accuracy as this corresponded to an error smaller than 0.3 cm² for the square shape which was closest in size to the hen's mean comb area.

We performed 3 types of precision analyses of PBA using the images of the hens’ combs by applying the CCC for repeated measures using the variance estimates of linear mixed models with hen as a random term (R Package cccrm v. 2.1.0) (Carrasco et al., 2013). First, to assess within-image precision, we selected one image per hen of the first 6 hens, using the same handler, with all hens laying on the side of their natural drop direction. For these 6 images we extracted PBA 3 times per image (N = 18). Then we calculated a CCC (function “cccUst”) across the repeatedly measured images.

Second, to analyze within-individual hen precision, we used the 6 natural drop images of all 15 hens (i.e., 2 handlers with 3 repeats per handler) (N = 90). Handler was included as a fixed factor in the mixed model for the CCC (function “cccvc”). As an additional estimate of within-individual variation the average range of estimated comb area was calculated across individuals, with individual ranges measured as max(combarea)−min(combarea) per hen.

Third, to test the effect of the natural drop direction of the combs on the precision of the estimated comb area, we selected 2 images for each hen. In one image, the hen lay on the side of the natural drop direction and in the second image the hen lay on the opposite side. For both images the same handler was involved (N = 30). The side of the comb depicted in the image was included as a fixed factor for the CCC estimation (function “cccvc”). Additionally, we calculated the mean difference between the 2 drop sides of the comb (mean(sizenaturaldrop−sizeoppositeside)) across individuals to inspect whether there was a measuring bias towards one side when calculating PBA.

For the comparison of the 2 approximation methods, the LHA and PBA, we descriptively compared average comb area, calculated mean absolute difference in comb areas, and analyzed the agreement between the methods using CCC (“DescTools”). We assessed LHA and PBA for bias (i.e., over-/underestimation of comb area) and whether the bias remained constant or revealed a trend (i.e., increasing/decreasing bias with increasing/decreasing comb area). For the analysis of the bias, we calculated the absolute and relative difference between the LHA and PBA, (absolute: |LHA−PBA|, relative: $\frac{2(\text{LHA}\;-\;\text{PBA})}{\text{LHA}+\text{PBA}}$) and inspected a Bland-Altman plot of the absolute difference. To analyze trend of the bias we used 2 linear models, with the absolute and relative differences in comb area, respectively, as response variables. The average comb area between LHA and PBA ($\frac{\text{LHA}\;+\;\text{PBA}}{2}$) was included as a covariate. Furthermore, we determined the rank order of hens based on comb area using LHA and PBA, respectively. We tested whether the 2 approximation methods achieved a similar rank order by calculating Spearman correlations between the rank orders.

Finally, we assessed how much the measures L and H contributed to the variation in PBA using a linear model. The response variable was the PBA value with L and H as covariates.

## RESULTS AND DISCUSSION

The validation of the pixel-based approximation resulted in high agreement between the approximated area of the geometric shapes and their actual areas (CCC > 0.99, 95% CI = [1, 1]) indicating high accuracy of the method. The mean absolute difference between the measured and known areas was small (mean difference = 0.18 cm², SD = 0.13 cm², max = 0.43 cm²), similarly to the MAPE (0.61%, SD = 0.5%, max = 1.54%), showing low measurement error.

The precision of PBA was excellent within-image (CCC = 0.99, 95% CI = [0.99, 1]) and within-individual hens (CCC = 0.99, 95% CI = [0.98, 1]). The average range of PBA within hens was 1.33 cm² (SD = 0.57 cm², max = 2.86 cm²), indicating low (4% of the area) within-individual deviation of comb area (mean comb area = 30.80 cm^2^, 1.33 cm²/30.80 cm² = 0.04). Values of PBA had substantial agreement between the 2 sides of the comb (CCC = 0.99, 95% CI = [0.97, 1]) and the mean absolute difference between the sides was 0.38 cm² (SD = 0.91 cm², max = 1.95 cm²). Thus, the natural drop direction did not appear to affect the precision. Comb areas seemed not to change over the course of the 5-min handling event, as one of the precision evaluations was performed on the first and last image of each hen and showed substantial agreement. The results of the validation provided evidence that PBA was an accurate and reliable estimate.

**Figure 2:**
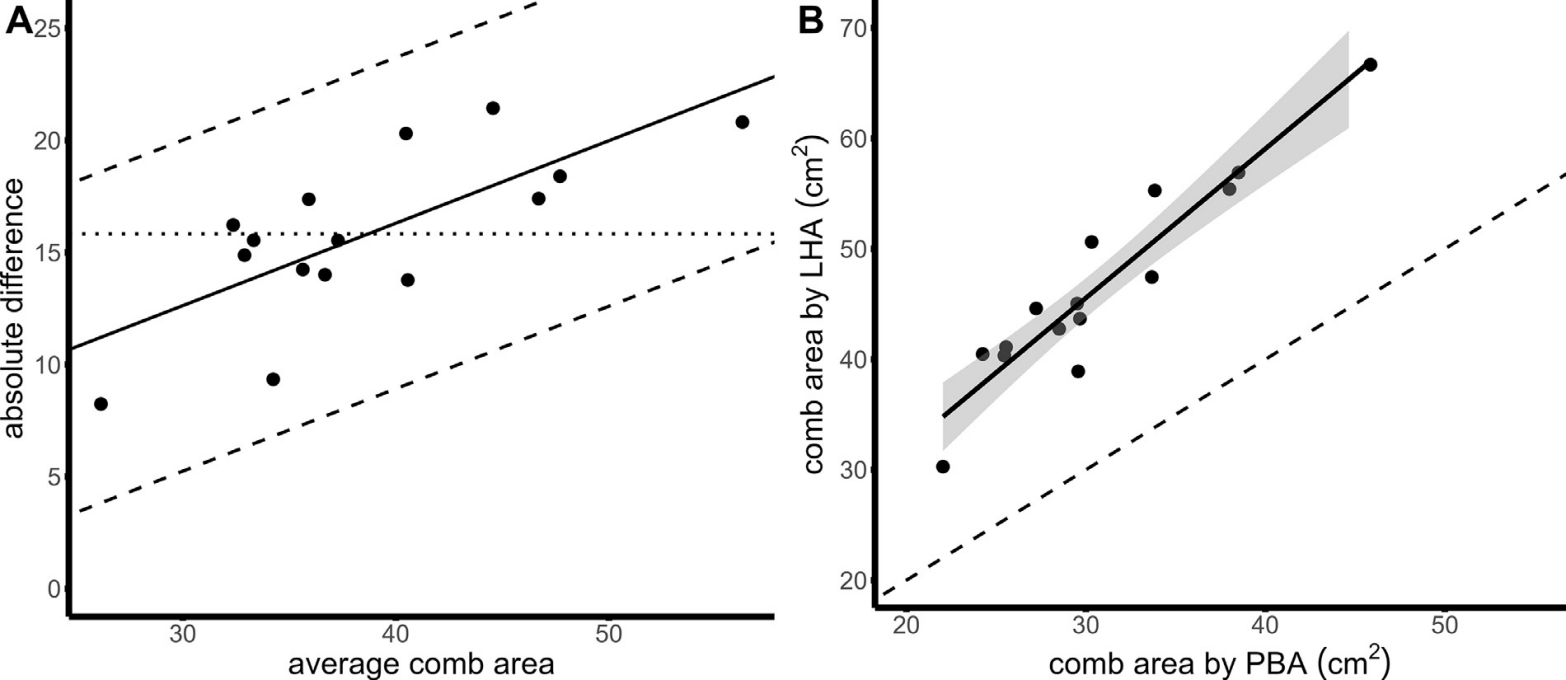
Results of the comparison between 2 comb area approximation methods. The figures compare approximations of comb area based on pixel-counts (PBA) and the product of the longest (L) and highest (H) dimensions of the comb (LHA). Each dot represents one chicken in both graphs. In panel A, a Bland–Altman plot is depicted. The *x*-axis shows the average comb area calculated between LHA and PBA. The *y*-axis indicates the absolute difference between comb areas from the 2 approximations, (LHA–PBA). The solid line is the mean difference in the approximations, corrected for the linear trend using a regression of *y*∼*x*, which shows the trend for an increase in difference with increased comb area. The dotted line shows the uncorrected mean difference (i.e., the bias). The dashed lines indicate the limits of agreement (i.e., 95% of differences would be expected within this range), corrected for the linear trend. Shown in B are the values of PBA and LHA for each individual hen. On the *x*-axis is the PBA, on the *y*-axis the LHA. The line shows a regression of *y*∼*x* with 95% confidence interval. The black, dashed line indicates a reference line of no difference between approximations.

When comparing the agreement of PBA and LHA, LHA (mean comb area = 46.63 cm², SD = 9.03 cm²) was on average 1.5 times greater than the PBA (mean comb area = 30.80 cm², SD = 6.29 cm², mean absolute difference in methods = 15.83 cm², SD = 3.77 cm², max = 21.44 cm²) (see also Figures 2A and 2B). Consequently, there was also poor agreement between PBA and LHA (CCC = 0.27, 95% CI = [0.11, 0.43]). Although the direct measures are traditionally more common and may even be considered a gold standard, the greater values of the LHA likely reflected the expected overestimation bias. As the LHA assumes a rectangular shape, whereas the PBA is based on pixels associated with actual comb area, the PBA is justifiably a more valid measure. We additionally detected a linear trend for the absolute difference between the 2 approximations to increase by 0.37 cm² (95% CI [0.17, 0.57]) with every unit increase of the relative comb area (F_1,13_ = 15.47, p = 0.002) (Figure 2A). The pattern suggests that the LHA overestimated larger combs more than smaller combs, resulting in over-proportioned representations of large combs compared to small combs. In other words, the LHA cannot reliably quantify the differences between comb areas (“how much bigger”) as it does not reflect the true difference between hens.

In contrast, the relative difference did not reveal an increase or decrease with comb area (β = −0.0003, 95% CI [−0.006, 0.005], F_1,13_ = 0.01, *P* = 0.91), indicating that both the overestimation bias and the linear trend of the LHA could be corrected. However, the relative difference ranged from 0.27 to 0.50 (mean relative difference = 0.41), implying a range of uncertainty of 0.23. Applied to our sample population with a mean comb area of 30 cm², such an uncertainty means an error margin of 7 cm². An error margin higher than the measured mean difference in comb area between individuals (6.6 cm², based on PBA) renders the results of a correction highly uncertain.

Nevertheless, the results of both approximations were highly correlated in rank order of hens based on comb area (r = 0.89, *P* < 0.001), indicating that the quality of comb area differences (“which is bigger”) were similar between approximations. Thus, we would recommend to evaluate the LHA on an ordinal scale level only (for further details, see Houle et al., 2011).

When we assessed how much of the variation in the PBA was explained by the 1-dimensional measures of L and H, respectively, the results showed a positive relationship between L and comb area (β = 4.29, 95% CI 1.41, 7.16], F_1,12_ = 79.33, *P* < 0.001), but no evidence of such a relationship with H existed (β = 4.25, 95% CI [−1.08, 9.59], F_1,12_ = 3.01, *P* = 0.11). These findings implied a greater contribution of the anterior-to-posterior dimension of a comb (L) to the variance in comb area in our study population than the inferior-to-superior dimension (H). Albeit the wide confidence intervals for both L and H highlighted the measurement uncertainty related to 1-dimensional measures of comb area.

A further aim was to assess whether the advantages of image-based methods could outweigh the disadvantages in application and thus replace direct methods. Disadvantages include requirements to take standardized pictures and image processing, which worsens with the number of assessed individuals and repetitions but could be improved by automation. The invasiveness of the method (catching, crating, handling) must be acknowledged, and prior habituation in case of repeated measures over several sessions should be performed to lower the manipulation stress. However, this is the case for both PBA and direct methods using calipers. To this date, there are no noninvasive methods to estimate chicken comb size on live hens, though future efforts could focus on extrapolating comb area from images without prior handling.

While the validation presented here was strictly performed with a female population, we would assume improved results for PBA when measuring cockerels due to larger comb sizes resulting in higher between-individual variation. In contrast, LHA would be expected to perform worse as the larger combs would be subjected to a stronger overestimation bias.

Considering the demonstrated superior accuracy and further advantages of PBA in comparison to LHA, we propose PBA be used for estimating comb area in future studies whenever possible. As such, PBA may allow future research to detect more subtle changes over time in comb size due to changes in androgen concentration or stress-related events.
